# Algicidal activity of *Streptomyces* sp. LMJ-114 against *Microcystis aeruginosa*

**DOI:** 10.3389/fmicb.2025.1669970

**Published:** 2025-11-12

**Authors:** Mijia Du, Qian Xie, Hongqiu Shi, Jianyuan Yang, Qigen Guo, Yuqin Zhang, Binghuo Zhang

**Affiliations:** 1 College of Pharmacy and Life Sciences, Jiujiang University, Jiujiang, China; 2 Institute of Medicinal Biotechnology, Chinese Academy of Medical Sciences & Peking Union Medical College, Beijing, China

**Keywords:** cyanobacterial blooms, *Microcystis aeruginosa*, *Streptomyces*, algicidal activity, microcystins, retinoic acids

## Abstract

Cyanobacterial blooms have become a worldwide problem. Chemical algicides play important role in controlling cyanobacterial blooms in spite of their potential secondary pollution to aquatic environments. The algicidal microorganisms and their metabolites are potential substitutes for non-selective chemical algicides because of their environmentally friendly characteristics. In this paper, an actinomycete strain, designated as LMJ-114, capable of eliminating cyanobacteria, was isolated from a soil sample collected from Lushan Mountains of China. Strain LMJ-114, belonging to *Streptomyces*, showed the highest similarity to *Streptomyces jiujiangensis* JXJ 0074^T^ based on the 16S rRNA gene sequences. This strain showed algicidal activities on both *Microcystis* and filamentous cyanobacteria. The extracellular water-soluble substances exhibited strong algicidal activity on *Microcystis aeruginosa* FACHB-905. The algicidal components from strain LMJ-114 could induce *M. aeruginosa* to produce massive reactive oxygen species (ROS), and seriously affected its antioxidant system. Lipid peroxidation, therefore, occurred seriously in cells of *M. aeruginosa*, which resulted in 91.6–525.2% higher of malondialdehyde (MDA), and disintegration of gelatinous sheaths, and subsequent sunk cell surface and perforation of the cells. The treatments of both culture broth supernatant and mycelia of strain LMJ-114 significantly affected the contents of retinoic acids (RAs) and microcystins (MCs), and 4 days later, RAs eliminated completely, and 7 days later, microcystin RR (MC-RR) decreased by 96.7 and 87.9%, respectively, and microcystin LR (MC-LR) contents decreased by 89.9 and 81.0%, respectively. L-valine was one of the algicidal compounds in the culture broth supernatant of strain LMJ-114. Strain LMJ-114 and its extracellular metabolites showed potential application in controlling cyanobacterial blooms.

## Highlights

- Cyanobacterial blooms are persistent environmental problems.
- An algicidal *Streptomyces* strain LMJ-114 was isolated from soil sample.
- LMJ-114 resulted in abundant ROS, and serious lipid-peroxidation in *M. aeruginosa* cells.
- LMJ-114 greatly reduced MCs and RAs in *M. aeruginosa* culture.

## 1 Introduction

Cyanobacterial blooms have been one of the prominent environmental concerns worldwide (Chen et al., 2011; de Figueiredo et al., 2004), especially in China (Guo, 2007; Wu et al., 2011). One of the most detrimental effects of cyanobacterial blooms is the production of large quantities of cyanotoxins. These toxins pose serious threats to the environment and human health (Azevedo et al., 2002). It is also a major reason for huge economic losses especially in aquaculture and fishing industries (Mizuno et al., 2008). An unforgettable tragedy caused by exposure to cyanotoxins occurred in 1996 in Caruaru, Brazil, during which 116 people became ill and 52 died of toxic hepatitis (Azevedo et al., 2002).

Cyanobacterial blooms are generally favored by high temperature and eutrophication (Matthijs et al., 2012). As such, cyanobacteria form the dominant phytoplankton during summer months (Contardo-Jara et al., 2008). Soaring agricultural and industrial activities without proper water management (de Figueiredo et al., 2004) and high input of nutrient-enriched pollutants into the aquatic environment are the major reasons for increasing eutrophication (de Figueiredo et al., 2004; Contardo-Jara et al., 2008), which further result in frequent breakouts of cyanobacterial blooms (de Figueiredo et al., 2004; Žegura et al., 2011). In tropical climates, blooms can occur throughout the year (Dejenie et al., 2009). Meanwhile, human activities have also accelerated the process of global warming (Davis et al., 2009; Paerl et al., 2008), which in turn promotes the growth of toxic cyanobacteria (Guo, 2007; Davis et al., 2009), leading to blooms with higher cyanotoxins content (Davis et al., 2009).

Another consequence of cyanobacterial blooms in eutrophic water is the presence of retinoic acids (RAs) and other analogues of retinoids. Various kinds of deformities can be generated in animals when their embryos are exposed to exogenous RAs (Gardiner et al., 2003). Cyanobacterial blooms in Taihu Lake have been found to produce high concentrations of RAs. 22 of 24 Cyanophyta present produce RAs and 4-oxo-RAs, the highest producer being *Microcystis flos-aquae* and *Microcystis aeruginosa* (Wu et al., 2012). These findings further reveal the serious detriment of cyanobacterial blooms. It is therefore urgently required to study effective means of eliminating the harmful cyanobacteria and their toxic metabolites from the water environments.

Many methods, such as biomanipulation, physical and chemical agents, have been employed to control cyanobacterial blooms. But all these methods have their drawbacks. For instances, the physical methods are usually expensive (Matthijs et al., 2012) and are applicable only in small impoundments such as pond and water reservoirs (Wu et al., 2011; Matthijs et al., 2012); chemical agents cause secondary pollution (Yan et al., 2011) though hydrogen peroxide can selectively kill the cyanobacteria without major impacts on other organisms in small lake (Matthijs et al., 2012), while application of biomanipulation in a large water ecosystem is difficult (Liu, 2010). Microbes and their metabolites show great potential in controlling cyanobacterial blooms. Algae-lysing microbes include virus, bacteria, actinomycetes and fungi (Sigee et al., 1999), and their main action modes are: (1) lysing the cyanobacterial cell after invading them (direct cyanobacterial cells inhibition), e.g., *Saprospira* sp. SS98-5 (Furusawa et al., 2003); (2) secreting bioactive compounds that lyse algae (indirect cyanobacterial cells inhibition), e.g., *Streptomyces eurocidicus* JXJ-0089 (Zhang et al., 2016b), and *S. jiujiangensis* JXJ 0074^T^ (Zhang et al., 2015, 2016a); (3) inducing production of self-cell lytic compounds when co-culturing with cyanobacteria, e.g., *Brevibacillus* (Ozaki et al., 2008).

In this study, the screening of algicidal activities and characterization of an algicidal *Streptomyces* sp., LMJ-114 is reported. The lytic properties of the strain and its metabolites, including the efficiency and algicidal range, the effects on *M. aeruginosa* physiology, and one of the algicidal compounds were also reported.

## 2 Materials and methods

### 2.1 Cyanobacteria culture

The cyanobacteria used in this study were all obtained from Institute of Hydrobiology, Chinese Academy of Sciences, and cultured in HGZ medium (Zhang et al., 2015) under an illumination of 35 μmol photon/m^2^/s on a 12 h light–dark cycle at 25 °C. *M. aeruginosa* FACHB-905 lawn was prepared as described below: 100 mL of algal culture (2–4 × 10^7^ CFU/mL) was mixed with 150–200 mL HGZ agar medium (cooling down to about 44 °C before mixing), and the mixture was poured into petri dishes and cultured at the condition described above. The algal lawns can be used for algicidal test after it become bright green.

### 2.2 Isolation and identification of algicidal strain LMJ-114

Actinomycete strains, isolated from soil samples collected from the Lushan Mountains of China by a serial dilution technique on ISP2 medium (yeast extract-malt extract agar) (Shirling and Gottlieb, 1966). The colonies growing on the ISP2 medium were placed on the algal lawns to test for the algicidal activity. The vanish of the bright green around the colony indicated that the strain has the algicidal activity. The morphological characteristics of algicidal actinomycete were observed by using a scanning electron microscopy (VEGA\\TESCNA) after the strain being cultured on ISP2 medium at 28 °C for 3–7 days. Extraction of genomic DNA was extracted using Rapid Bacterial Genomic DNA Isolation Kit (Sangon Biotech, Shanghai, China) according to the instructions of the manufacturer. The 16S rRNA gene sequence was amplified using universal bacterial primers 27F (5’-AGAGTTTGATCMTGGCTCAG-3′) and 1492R (5’-TACGYTACCTTGTTACGACTT-3′).

### 2.3 Algicidal activity of strain LMJ-114 and its different metabolites

Algicidal strain was cultured using ISP2 liquid medium (No agar) on a shaker at 28 °C and 120 r/min for 6 days. The culture broth was centrifuged at 4527 × g for 10 min with the supernatant and the mycelial precipitate being collected, respectively. The supernatant was then treated by reduced pressure distillation at 50 °C, and the resultant solid material was fully extracted using ethyl acetate and deionized water successively to obtain the extracellular fat- and water-soluble metabolites of strain LMJ-114. The mycelial precipitate was firstly immersed in a mixture of ethyl acetate-methyl alcohol-deionized water (1:1:1, vol/vol/vol) at 50 °C to release its intracellular metabolites into the mixture. Then the soaking extract solution was treated as described above to obtain the intracellular fat- and water-soluble metabolites of strain LMJ-114. Algicidal assay was performed using the culture broth supernatant, fat- and water-soluble extracts of intracellular and extracellular metabolites and mycelia of the strain with the experimental set-up described below: 2 mL of culture broth supernatant (filtered through 0.45 μm membrane filter), 10 mg each of fat- and water-soluble extracellular and intracellular metabolites, 0.5, 1.0, 1.5 and 2.0 g of mycelia, were added into separate 100 mL of *M. aeruginosa* FACHB-905 culture (1 × 10^7^ CFU/mL). Nothing was added into 100 mL of *M. aeruginosa* FACHB-905 culture (1 × 10^7^ CFU/mL) for the control group. They were incubated for 3 days and the algicidal activities were determined by the removal rates of the chlorophyll *a* (chl-*a*) content. The chl-*a* contents were measured using hot-ethanol extract method (Chen et al., 2006) as following steps: (i) the algae cultures were sampled and the samples were centrifuged at 4527 × g for 10 min; (ii) the resultant algal cells precipitates were stored at −20 °C for 24 h, and then mixed with 5 mL hot-ethanol of 90% (85 °C) and heated using water bath of 85 °C for 1 min, and kept in dark place at room temperature for 4–6 h to extract the chl-*a*; (iii) the mixtures were centrifuged at 4527 × g for 10 min, and the absorbances (E_665_ and E_750_) of the resultant supernatants were measured at 665 and 750 nm, respectively, using ethanol of 90% ethanol as the reference for zero adjustment; (iv) the absorbances (A_665_ and A_750_) of the samples were measured at 665 and 750 nm once again, respectively, after being treated with hydrochloric acid solution (1 M; 70 μL for 3 mL supernatant) for 1 min. Chl-*a* contents were calculated by the formula: Chl-*a* content (mg/L) = 27.9 × V_e_ × (E_665_ − E_750_ − A_665_ + A_750_)/*V_s_*, where, V_e_ and *V_s_* represent the volumes (mL) of ethanol used to extract chl-*a* and algae culture samples collected, respectively.

### 2.4 Effect of metabolites of LMJ-114 on the morphology of *Microcystis aeruginosa*

After being treated with the culture broth supernatant of strain LMJ-114 (culture broth supernatant of strain LMJ-114 was abbreviated to CBSL) for 2 days, *M. aeruginosa* FACHB-905 was centrifuged at 2800 × g for 10 min at 4 °C, and the resultant cell precipitate was treated with 0.1 M phosphate-buffered saline (PBS, pH 7.0) containing 2.5% glutaraldehyde for 1 h at 4 °C. After removing PBS, the algal cells were dehydrated for 4 min by successively using 50, 60, 70, 80, 90, and 100% ethanol, and dried on clean coverslip and coated with gold for scanning electron microscopy (VEGA\\TESCNA) analysis.

### 2.5 Antioxidant system assays of *Microcystis aeruginosa* FACHB-905

Three percent (vol/vol) of CBSL (replaced with 3 mL HGZ medium for control group) were added into *M. aeruginosa* FACHB-905 culture (2.0 × 10^7^ CFU/mL), and cultured under the condition described above. Then the cultures were sampled every 12 h, and the samples were centrifuged at 4527 × *g* for 20 min at 4 °C. The algae cell precipitates were homogenized using an ultrasonic cell pulverizer (JY92-2DN; Xinzhi Co., Ningbo, China) at 200 W for 5 min (ultrasonic time, 2 s; rest time, 8 s) under ice bath cooling. Then, the homogenates were centrifuged at 12,000 g for 10 min at 4 °C. The resultant supernatants were used to detect the lipid-peroxidation, enzymatic and non-enzymatic antioxidant of *M. aeruginosa*.

The activities of superoxide dismutase (SOD; EC 1.15.1.1), catalase (CAT; EC 1.11.1.6) and peroxidase (POD; EC 1.11.1.7) were measured according to the method described by Chen and Wang (2006). For measuring the SOD activity, the reaction mixture contained 1.9 mL PBS solution (50 mmol/L, pH7.8, containing 100 μmol/L EDTA), 0.3 mL methionine solution (220 mmol/L), 0.3 mL nitroblue tetrazolium (NBT) solution (1.25 mmol/L), 0.3 mL riboflavin solution (33 μmol/L), and 0.2 mL cell-free extract (replaced with 0.2 mL PBS for both negative control and positive control). Both treatment and positive control were exposed to an illumination of 70 μmol photon/m^2^/s for 20 min at 25 °C, and the negative control was kept wrapped in aluminum foil to prevent any photochemical reaction of NBT. And then the absorbances of treatment and positive control were measured at 560 nm after adjusting zero using negative control. One unit (U) of SOD activity is defined as the amount of enzyme required to inhibit 50% of photochemical reactions of NBT. The specific activity of SOD (SOD_SA_) was calculated according the following formula: SOD_SA_ (U/mg) = V_E_ × (A_P_ − A_T_) / (0.5 × A_P_ × V_S_ × 0.2 × C_P_), where V_E_ and V_S_ were volumes (mL) of the cell free extracts and algal samples collected, respectively; and A_P_ and A_T_ were the absorbances of the positive controls and treatments, respectively; C_P_ (mg/mL) was the protein content of the cell free extract. The protein contents in the cell free extracts were determined by using method of Coomassie brilliant blue G-250, in which bovine serum albumin was used as the standard protein.

For measuring CAT activity, the reaction was done in a total volume of 3 mL, containing 1.8 mL PBS solution (50 mmol/L, pH 7.0), 1.0 mL H_2_O_2_ (0.1 mol/L) and 0.2 mL cell-free extract. The absorbance decreases of the mixtures in 5 min were measured at 240 nm. One unit (U) of CAT activity is defined as the decrease in absorbance at 240 nm by 0.01 in 1 min. The specific activity of CAT (CAT_SA_) was calculated according the following formula: CAT_SA_ (U/mg) = (D_A_ × V_E_) / (0.2 × V_S_ × 5 × 0.01 × C_P_), where D_A_, V_E_ (mL), V_S_ (mL), and C_P_ (mg/mL) were the decrease values of the absorbance, total volume of the cell free extracts, volume of the algal samples collected, and protein content of the cell free extract. The protein contents were measured as described above.

The reaction mixture for POD assay contained 1.0 mL PBS solution (50 mmol/L, pH7.0), 1.0 mL guaiacol (16 mmol/L), 1.0 mL H_2_O_2_ (0.1 mol/L), and 1.0 mL cell-free extract. The absorbance increases of the reaction mixtures in 5 min were measured at 470 nm. One unit (U) of POD activity is defined as the increase in absorbance at 470 nm by 0.01 in 1 min. The specific activity of POD (POD_SA_) was calculated according the following formula: POD_SA_ (U/mg) = (I_A_ × V_E_) / (1 × V_S_ × 5 × 0.01 × C_P_), where I_A_, V_E_ (mL), V_S_ (mL), and C_P_ (mg/mL) were the increase values of the absorbance, total volume of the cell free extracts, volume of the algal samples collected, and protein content of the cell free extract. The protein contents were measured as described above.

The assay for ascorbic acid (AsA) content was based on the formation of the red complex between 2,2′-bipyridine and ferrous ion (reduced from ferric ion by AsA in acid solution) (Chen and Wang, 2006). 0.3 mL each of cell-free extract, NaH_2_PO_4_ (150 mmol/L, pH7.4) and H_2_O were mixed, followed by addition of 0.6 mL trichloroacetic acid (TCA, 600 mmol/L), 0.6 mL H_3_PO_4_ (4.49 mol/L), 0.6 mL 2,2′-bipyridine (250 mmol/L) and 0.3 mL FeCl_3_ (185 mmol/L) after 30 s. The reaction mixture was incubated at 37 °C for 60 min and the absorbance was measured at 525 nm. The AsA content of the cell-free extract was calculated according AsA standard graph: C_AsA_ (μmol/mL) = (A_525_−0.001)/1.4409, where A_525_ was the absorbance of the reaction mixture at 525 nm. AsA standard graph was drawn as described above using different concentrations of AsA.

The content of reduced glutathione (GSH) was determined by measuring the formation rate of 5-thio-2-nitrobenzoic acid (TNB) from 5,5′-dithiobis-(2-nitrobenzoic acid) (DTNB) (Anderson, 1985). The reaction mixture includes 0.25 mL cell-free extract, 2.6 mL NaH_2_PO_4_ (150 mmol/L, pH 7.7), and 0.15 mL DTNB (6 mmol/L). After incubation at 30 °C for 5 min, the absorbance of the solutions was measured at 412 nm. The GSH content of the cell-free extract was calculated according GSH standard graph: C_GSH_ (μmol/mL) = (A_412_−0.0003)/1.3212, where A_412_ was the absorbance of the reaction mixture at 412 nm. GSH standard graph was drawn as described above using different concentrations of GSH.

Lipid peroxidation was determined by measuring the content of malondialdehyde (MDA) according to the method described by Chen and Wang (2006). 2 mL cell-free extract and 2 mL thiobarbituric acid reagent (0.5% in 10% TCA) were mixed and heated for 20 min at 100 °C. The mixture was cooled and centrifuged at 4527 × *g* for 20 min at 4 °C. The absorbances of the supernatants were measured at 450 nm, 532 nm and 600 nm, respectively. MDA content was calculated by the following formula: C_MDA_ (μmol/L) = 6.45 (A_532_ − A_600_) − 0.56 × A_450_, Where A_450_, A_532_, and A_600_ represents the absorbances of the supernatants at 450 nm, 532 nm and 600 nm, respectively.

### 2.6 Algicidal range of CBSL

Three milliliters of CBSL were added into the following cyanobacteria strains of 100 mL, and cultured for 3 days as described above. Then the chl-*a* contents of these cyanobacteria were measured as described above. *M. aeruginosa* FACHB-905, *Microcystis wesenbergii* FACHB-1112, *Microcystis viridis* FACHB-1284, *Aphanizomenon flos-aquae* FACHB-1171, *Oscillatoria planctonica* FACHB-708, and *Anabaena flos-aquae* FACHB-1092 were involved in this study. In control groups, 3 mL HGZ medium replaced 3 mL CBSL in the above cyanobacterial cultures.

### 2.7 Effect of strain LMJ-114 on the contents of microcystins and RAs

*Microcystis aeruginosa* FACHB-905 produces high contents of microcystins (MCs) (Zhang et al., 2016b) and RAs (Wu et al., 2012), and therefore it was used in this study. The RAs and MCs contents were detected after 4 and 7 days of cultivation, respectively. *M. aeruginosa* FACHB-905 culture (2 × 10^7^ CFU/mL, 100 mL) were treated with 2 mL of CBSL and 2 g mycelial cake (0.23 g dry weight) of strain LMJ-114. The cultures were centrifuged at 4527 × *g* for 20 min at 4 °C. Both the supernatants and sediments were collected for analysis of MCs and RAs.

MCs in the supernatants were extracted using solid-phase extraction (SPE) columns (Bakerbond™ spe 7020-06, Octadecyl C_18_ Disposable Extraction Columns; J. T. Baker, United States) as described below: the SPE columns were pretreated with 5 mL methanol in 0.1% (v/v) trifluoroacetic acid (TFA), 5 mL 100% methanol and 10 mL water; and then the supernatants were loaded into the SPE columns, the toxins were eluted with 8 mL methanol in 0.1% (v/v) TFA after the SPE columns being washed by 5 mL of 5, 10 and 20% methanol successively. The eluent was evaporated to dryness, and redissolved in 1.0 mL deionized water for analysis by HPLC. The sediment was mixed with 30 mL of methanol/water (75:25, vol/vol), and homogenized in an ice bath, and centrifuged at 4527 × *g* for 20 min at 4 °C. The supernatant was collected, while the residue was retreated as described above. The supernatants obtained were combined and evaporated to dryness. The dry residues were redissolved in 20 mL of deionized water, and the extracted intracellular toxins were purified by the method described above and analyzed by HPLC. MC-RR and MC-LR were analyzed using Agilent 1,200 LC with an Agilent LC C_18_ column (Eclipse XDB-C18, 5 μm, 4.6 × 150 mm). The mobile phase in channel A was water containing 0.05% (v/v) TFA and the mobile phase in channel B was acetonitrile containing 0.05% (v/v) TFA. Elution was performed with 10–60% B linear gradient for 20 min at a flow rate of 1 mL/min. The absorbance was monitored at 240 nm with sample injection volumes of 20 μL.

RAs were quantified according to Wu et al. (2010, 2012) with some modifications. The Oasis HLB cartridge (6 mL, 200 mg, Waters) was pretreated with 6 mL ethyl acetate, 6 mL methanol, and 12 mL ultrapure water, at a flow rate of 7 mL/min before the supernatant was loaded. The cartridge was then dried and followed by elution of the analytes with 7 mL ethyl acetate (containing 0.5% acetic acid) and 3 mL acetone. The extracted eluent was evaporated to dryness at 27 °C and the dry residues redissolved in 0.4 mL methylene chloride and 4 mL hexane. The solutions obtained were purified in a silica cartridge (6 mL, 500 mg, Waters; pretreated with 8 mL of hexane). After the cartridge was washed with 2 mL of hexane-methylene chloride (1:1, vol/vol), elution was done with 10 mL of hexane-methylene chloride-isopropanol-acetic acid (87:10:1:2, vol/vol/vol/vol). The extract was evaporated to dryness under the condition described above and redissolved in 0.25 mL methanol. The samples were filtered through 0.22 μm filter membrane before analyzing by HPLC. The sediments were mixed with 30 mL acetone and homogenized in an ice bath, followed by centrifugation at 4527 × *g* for 20 min at 4 °C. The supernatant was collected and the residue was treated again as described above. The combined supernatants were purified as the methods described above before analyzing by HPLC. The chromatographic mobile phases were water containing 0.5% (vol/vol) acetic acid in channel A and methanol containing 0.5% (vol/vol) acetic acid in channel B. The elution was done for 17 min with a flow rate of 1 mL/min in the mobile phase ratio of 14:86 (channel A: channel B, vol/vol). The absorbance was monitored at 353 nm with sample injection volumes of 50 μL.

### 2.8 Purification and elucidation of algicidal compounds

Water-soluble extracellular metabolites were purified repeatedly by column chromatography of Sephadex™ LH-20 (GE healthcare) and C_18_ (YMC*GEL, ODS-AQ-HG) using deionized water/methanol (1:1; vol/vol) and deionized water/methanol (1:0 → 0:1; vol/vol) as the elution solvents, respectively. The algicidal activities of the fractions were assayed by filter paper method using *M. aeruginosa* lawn (Zhang et al., 2016b). The algicidal compound was elucidated from the data of ^13^C NMR and ^1^H NMR (in D_2_O) obtained on a Bruker DRX-500 MHz instrument with tetramethylsilane (TMS) as the internal standard.

### 2.9 Toxicity of culture broth of LMJ-114 on aquatic organisms

The toxicity of strain LMJ-114 and its metabolites on aquatic organisms were studied by using *Carassius auratus* var. *pengzesis* (weighing approximately 25 g) and *Viviparus chinensis* (~2.87 g). One percent (vol/vol) culture broth of strain LMJ-114 was added into a fish tank (volumes maintained at 100 L for 50 fish, and 20 L for 120 viviparids). The energetic fish with few scales shed were selected for this test. The viviparids were collected from the pond in the wild field. The water and culture broth in the fishpond were replaced every 24 h. Fifty grams of rice and 20 grams of fresh vegetable leaves were supplied for fish and viviparids of a fish tank, respectively. The growth and mortalities of the test aquatic organisms were calculated 7 days later. No culture broth of strain LMJ-114 was added into the controls. These tests were carried out at 20 °C under laboratory conditions.

### 2.10 Data analysis

Statistical analyses were performed using the SPSS19.0 software. The mean value and standard deviation (SD) of three replicates were calculated, and the graph was plotted using the mean value. All error bars indicate the SD of three replicates. Comparisons of the activities of SOD, CAT, and POD, and the contents of chl-*a*, RAs, AsA, GSH, MDA, MC-RR, and MC-LR between treatments and controls were performed using analysis of variance (ANOVA) followed by Tukey’s pairwise comparisons. Significance was set at *p* values of 0.05.

## 3 Results

### 3.1 Isolation and characterization of algicidal strain LMJ-114

Strain LMJ-114 exhibited strong algicidal activity on *M. aeruginosa* FACHB-905 lawn, and it showed an inhibition zone diameter of about 3.6 cm against its colony diameter of 0.7 cm after 3 days of incubation (Supplementary Figure S1).

**Figure 1:**
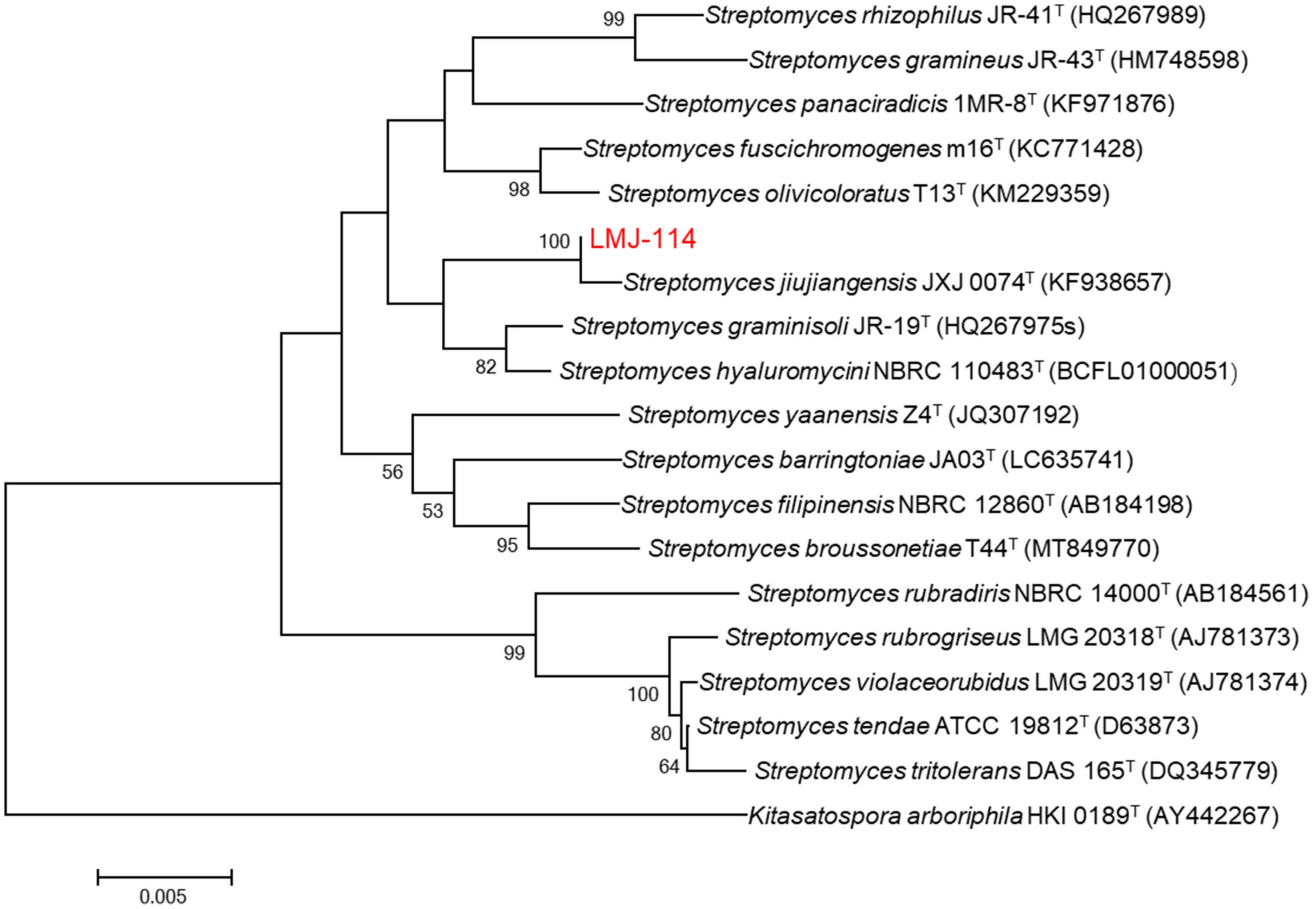
Neighbor-joining phylogenetic tree based on 16S rRNA gene sequences of strain LMJ-114 and its closest relative species of the genus *Streptomyces*. Bootstrap values (≥50%) based on 1,000 replications are shown at the branching points. Bar, 0.005 changes per nucleotide position.

Strain LMJ-114 developed well-branched substrate and aerial mycelia on ISP2 medium. At maturity, the aerial mycelia formed straight or spiral spore chains, and the spores were offwhite in color and ellipsoid in shape (Supplementary Figure S2). Its 16S rRNA gene sequence (1,446 bp; GenBank accession number: PV915256) showed the highest similarity to that of *Streptomyces jiujiangensis* 0074^T^ (99.51%), and they also formed a distinct clade on neighbor-joining phylogenetic tree with 100% of bootstrap value (Figure 1). Therefore, strain LMJ-114 belonged to the genus of *Streptomyces*.

### 3.2 Algicidal efficiency of strain LMJ-114 and its different metabolites

**Figure 2:**
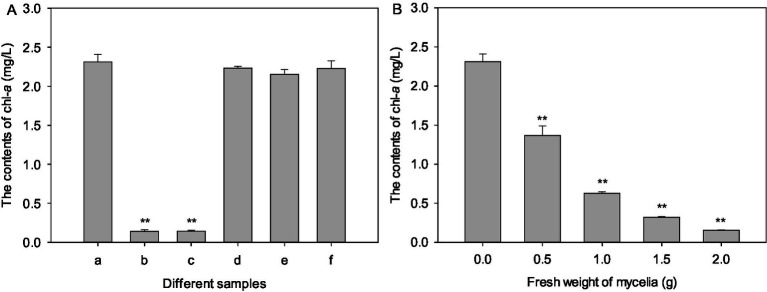
The algicidal activities of metabolites and mycelia of strain LMJ-114. **(A)** treated with different metabolites, **(B)** treated with mycelia. (a) Control, (b) culture broth supernatant, (c) water-soluble extracellular metabolites, (d) fat-soluble extracellular metabolites, (e) water-soluble intracellular metabolites, (f) fat-soluble intracellular metabolites. Error bars indicate standard deviations for the three replicates. Comparisons between control and different treatments were performed using ANOVA. Significant differences are shown by asterisks: **, *p* < 0.01.

The pH value of culture broth of strain LMJ-114 was about 7.0. And the pH value of algal culture was not affected by the addition of the culture broth of strain LMJ-114 in this study, which indicated that strain LMJ-114 produced algicidal metabolites. Chl-*a* content of the control increased initially from 1.63 mg/L to 2.31 mg/L after 3 days of cultivation. Both CBSL and extracellular water-soluble substances exhibited strong algicidal activity (Figure 2A), and the chl-*a* contents treated with both CBSL and extracellular water-soluble substances were only 0.14–0.141 mg/L, decreased by 94% than that of the control (*p* < 0.01). However, the chl-*a* contents treated with extracellular fat-soluble substances and intracellular components were similar to that of the control (*p* > 0.05), indicating that these components had no obvious algicidal activity on *M. aeruginosa* FACHB-905. The mycelia of strain LMJ-114 also showed strong algicidal activity on *M. aeruginosa* FACHB-905 with a dose effect (Figure 2B). The chl-*a* content in group treated with 0.5 g of mycelia were 1.37 mg/L, which was 40.7% lower than that of the control (*p* < 0.01); while the chl-*a* content in group treated with 2 g of mycelia were only 0.154 mg/L, 93.3% lower than that of the control (*p* < 0.01).

### 3.3 Morphological damage of cyanobacteria by CBSL

**Figure 3:**
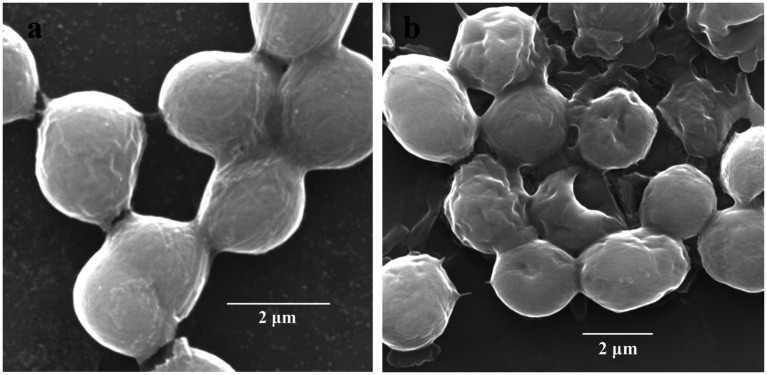
Scanning electron micrographs of *M. aeruginosa* FACHB-905 cells. **(a,b)** Represent algal cells of the control and the treatment of CBSL, respectively.

The cellular surfaces of *M. aeruginosa* FACHB-905 of the control group were intact and covered with gelatinous sheaths. However, the gelatinous sheaths of the algae cells treated with CBSL tended to be disintegrated, and its cell surfaces were sunken and perforated (Figure 3), indicating that the metabolites of strain LMJ-114 can damage the surface morphological structure of algal cells.

### 3.4 Effects on the antioxidant system of *Microcystis aeruginosa* FACHB-905

**Figure 4:**
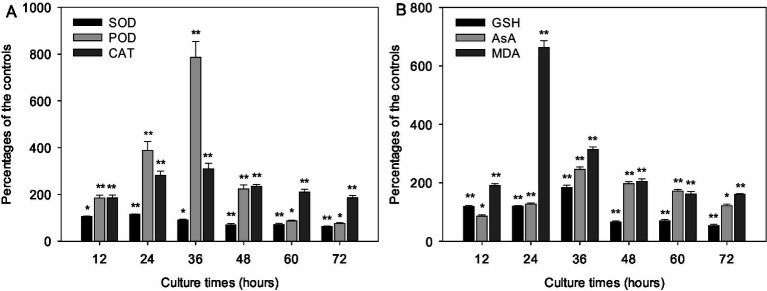
Effects of metabolites from strain LMJ-114 on the antioxidant system of *M. aeruginosa* FACHB-905. **(A)** antioxidant enzymes, **(B)** non-enzymatic antioxidants and MDA. Error bars indicate standard deviations for the three replicates. Comparisons between controls and treatments at different times were performed using ANOVA. Significant differences are shown by asterisks: *, *p* < 0.05; **, *p* < 0.01.

Metabolites from strain LMJ-114 severely affected the function of cyanobacterial antioxidant system. The activities of SOD, POD and CAT in groups treated with CBSL gradually increased initially from 31.3, 5.2, and 10.9 U/mg protein to their maximum values (33.9, 20.1 and 33.1 U/mg protein, respectively) at 24, 36 and 36 h, respectively, which were 15.6, 686.0, and 209.8% higher than those of the control groups (Figure 4A) (*p* < 0.01). And then their activities decreased quickly to 16.4, 1.8 and 18.9 U/mg protein, respectively, which were 37.3 and 24.2% lower and 85.7% higher than those of the control groups (Figure 4A) (*p* < 0.01), respectively. The contents of GSH, AsA, and MDA were also seriously influenced by CBSL (Figure 4B). The contents of GSH and AsA in test group treated with CBSL increased initially from 0.129 and 0.252 μmol/mg protein to 0.444 and 0.451 μmol/mg protein at 36 h, respectively, which were 83.5 and 146.0% higher than those of the controls (*p* < 0.01), respectively; and then decreased to 0.094 and 0.190 μmol/mg protein at 72 h, which was 46.8% lower and 21.6% higher than those of the controls (Figure 4B) (*p* < 0.01, *p* < 0.05), respectively. The MDA contents of the cell free extract of the controls were between 0.099–0.120 μmol/L during the test time. However, the MDA contents in the cell free extract of the test groups treated with CBSL increased initially from 0.120 μmol/L to 0.621 μmol/L at 24 h, 525.2% higher than that of the control (Figure 4B) (*p* < 0.01). Then the MDA contents of treatment decreased quickly to 0.224–0.234 μmol/L, which were still 98.9–252.2% higher than those of the controls (Figure 4B) (*p* < 0.01).

### 3.5 Effect of strain LMJ-114 on MCs and RAs contents

**Figure 5:**
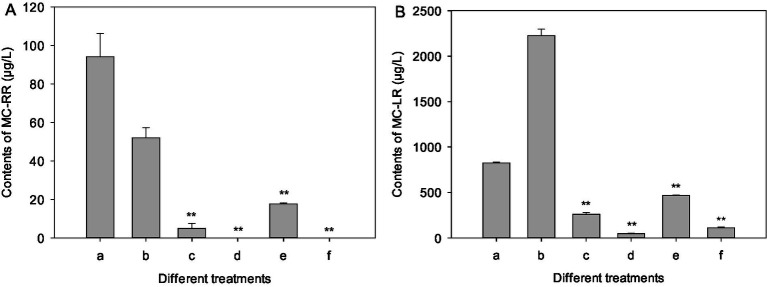
Influences of treatments of CBSL and mycelia on MC-RR and MC-LR contents of *M. aeruginosa*. **(A)** MC-RR, **(B)** MC-LR. (a) Extracellular MCs of the controls, (b) intracellular MCs of the controls, (c) extracellular MCs treated with mycelia, (d) intracellular MCs treated with mycelia, (e) extracellular MCs treated with CBSL, (f) intracellular MCs treated with CBSL. Error bars indicate standard deviations for the three replicates. Comparisons between controls and treatments were performed using ANOVA. Significant differences are shown by asterisks: **, *p* < 0.01.

The extracellular and intracellular MC-RR contents of the control increased initially from 20.9 and 14.7 μg/L to 94.1 and 52.0 μg/L after 7 days of culture (Figure 5A), respectively. However, the extracellular MC-RR contents of the test groups treated with mycelia and CBSL decreased initially from 20.9 μg/L to 4.9 and 17.6 μg/L 7 days later, respectively, which were 94.8 and 81.3% lower than that of the control (*p* < 0.01) (Figure 5A), respectively. And the intracellular MC-RR of the extracts treated with mycelia and CBSL were not detected (Figure 5A). After 7 days of culture, the extracellular and intracellular MC-LR contents of the control decreased and increased initially from 1301.7 and 1101.3 μg/L to 824.9 and 2224.2 μg/L, respectively. The extracellular MC-LR contents of the test groups treated with mycelia and CBSL were 261.0 and 468.6 μg/L 7 days later, which were 68.4 and 43.2% lower than that of the control (*p* < 0.01) (Figure 5B), respectively. And the intracellular MC-LR contents of the test groups treated with mycelia and CBSL were 47.7 and 112.2 μg/L 7 days later, which were 97.9 and 95.0% lower than that of the control (*p* < 0.01) (Figure 5B), respectively. After 4 days of culture, the extracellular and intracellular RAs contents of the control were 999.4 and 1292.4 ng/L, respectively. However, RAs could not be detected in the test groups treated with both mycelia and CBSL.

### 3.6 Algicidal compound from strain LMJ-114

The algicidal assays using different components isolated from the fermentation broth of strain LMJ-114 indicated that its metabolites comprised many algicidal compounds. And a white powder with strong algicidal activity on *M. aeruginosa* FACHB-905 lawn was purified. The algicidal compound showed no absorbance at 254 nm or 365 nm wavelength, but was stained by ninhydrin indicating that it is an amino-group containing compound. *S. jiujiangensis* JXJ 0074^T^ produces algicidal L-valine (Zhang et al., 2016a). Therefore, we detected both the algicidal compound isolated from LMJ-114 and the L-valine using thin layer chromatography of silica gel plate, and these two compounds had the same R_f_ values and ninhydrin staining (Supplementary Figure S3). The spectra of ^1^H NMR, ^13^C NMR, and DEPT (distortionless enhancement by polarization transfer) (in D_2_O) showed this compound has one carbonyl (*δ*_C_ 175.0), two methines (*δ*_C_ 61.1, *δ*_H_ 3.47; 29.8, *δ*_H_ 2.14), and two methyls (*δ*_C_ 18.7, *δ*_H_ 0.91; *δ*_C_ 17.3, *δ*_H_ 0. 85) (Supplementary Figure S4). These spectra data were similar to L-valine (Pretsch et al., 2000; Zhang et al., 2016a). Based on these data above, the algicidal compound from LMJ-114 was identified as L-valine.

### 3.7 Algicidal range of CBSL

**Figure 6:**
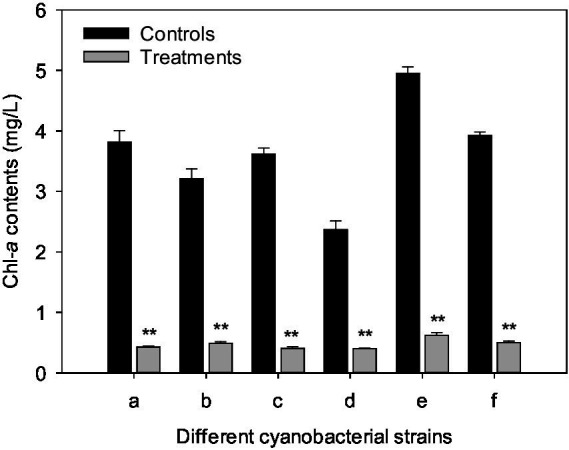
Influences of CBSL on six cyanobacterial strains. (a) *M. aeruginosa* FACHB-905, (b) *M. wesenbergii* FACHB-1112, (c) *M. viridis* FACHB-1284, (d) *Aphanizomenon flos-aquae* FACHB-1171, (e) *Oscillatoria planctonica* FACHB-708, (f) *Anabaena flos-aquae* FACHB-1092. Error bars indicate standard deviations for the three replicates. Comparisons between controls and treatments were performed using ANOVA. Significant differences are shown by asterisks: **, *p* < 0.01.

After 3 days of culture, the chl-*a* contents of *M. aeruginosa* FACHB-905, *M. wesenbergii* FACHB-1112, *M. viridis* FACHB-1284, *Aphanizomenon flos-aquae* FACHB-1171, *Oscillatoria planctonica* FACHB-708, and *Anabaena flos-aquae* FACHB-1092 were 3.82, 3.21, 3.62, 2.37, 4.95, and 3.92 mg/L for the controls, respectively. However, after treated with CBSL, the chl-*a* contents of six cyanobacterial strains were 0.402–0.621 mg/L, which were 83.0–88.8% lower than these of the controls (*p* < 0.01) (Figure 6).

### 3.8 The toxicity on aquatic organisms

All fish grew well and no fish died for both control group and test group during the test period, indicating that culture broth of strain LMJ-114 was not toxic to fish. Meanwhile, twenty viviparids died in control group, which accounted for 5.56% of 360 viviparids. And nineteen viviparids died in treatment group, which accounted for 5.28% of 360 viviparids, similar to that of the control group (*p* > 0.05). Therefore, culture broth of strain LMJ-114 was also not toxic to mollusk viviparid.

## 4 Discussion

Strain LMJ-114, a member of *Streptomyces*, showed strong algicidal activity on both unicellular *Microcystis*, such as *M. aeruginosa* FACHB-905, *M. wesenbergii* FACHB-1112, and *M. viridis* FACHB-1284, and filamentous cyanobacteria, such as *Aphanizomenon flos-aquae* FACHB-1171, *Oscillatoria planctonica* FACHB-708, and *Anabaena flos-aquae* FACHB-1092 (Figure 6). Its metabolites resulted in abundant ROS, and serious lipid-peroxidation in *M. aeruginosa* cells. L-valine is one of its algicidal compounds.

*S. jiujiangensis* JXJ 0074^T^ showed the highest similarity to strain LMJ-114. However, the supernatant of its culture broth has strong algicidal activity mainly on *Microcystis* such as *M. aeruginosa* FACHB-905, *M. aeruginosa* FACHB-1203, *M. wesenbergii* FACHB-1112, *M. viridis* FACHB-1284 and *M. flosaquae* FACHB-1285, and weak or no activity on filamentous cyanobacteria, such as *Oscillatoria planctonica* FACHB-708, *Anabaena flosaquae* FACHB-1092, *Nostoc punctiforme* FACHB-252 and *Oscillatoria tennuis* FACHB-247 (Zhang B. et al., 2014). Strain JXJ 0074^T^ produced algicidal compounds L-valine and 2′-deoxyguanosine. The former showed algicidal activity mainly on *Microcystis* (Zhang et al., 2016a), and the latter showed low to moderate algicidal activity on *Microcystis* and filamentous cyanobacteria at 10 μg/mL (Zhang et al., 2015). Therefore, strain LMJ-114 can probably produce other unknown algicidal compounds that different from these produced by *S. jiujiangensis* JXJ 0074^T^. Furthermore, different media were used in the fermentations of *S. jiujiangensis* JXJ 0074^T^ (Zhang B. et al., 2014) and LMJ-114, which was probably another reason that the metabolites from the two strains showed different algicidal activity on filamentous cyanobacteria. More study should be performed to illustrate this question.

The formation of reactive oxygen species (ROS) is accelerated under stress conditions (Noctor and Foyer, 1998). ROS, including ·OH, O_2_^−^, H_2_O_2_, and ^1^O_2_, can initiate free radical reactions in biological cell, which causes significant damages to organism. Chain reactions are one of the most significant characteristics of radical reactions. O_2_^−^ is the most active reactive oxygen except ·OH. SOD can terminate O_2_^−^ initiating chain reaction by converting it into O_2_ and H_2_O_2_. Algicidal compounds from *Streptomyces* can result in a large amount of O_2_^−^ (more 240% higher than that of the control) in cyanobacterial cells (Zhang et al., 2015, 2016a, 2016b). And upon exposure to CBSL, the cyanobacterial SOD activity only increased to 115.6% of the control after 24 h of culture, and then decreased quickly to only 62.7% of the control after 72 h of culture (Figure 4A). As a result, most of the O_2_^−^ induced by CBSL cannot be terminated by SOD. Lipid-peroxidation is a typical radical chain reaction which can be easily initiated on the biological membrane as there are plenty of oxygen and polyunsaturated fatty acids. Therefore, the residual O_2_^−^ can initiate a large amount of lipid-peroxidation, which was probably the main reason that MDA contents were far higher than these of the controls during the test (Figure 4B).

H_2_O_2_ can be decomposed into nontoxic H_2_O and O_2_ by CAT and POD. Two important antioxidative compounds AsA and GSH serve as substrates for POD (Noctor and Foyer, 1998), and also help in H_2_O_2_ detoxification (Mehlhorn et al., 1996). Upon exposure to CBSL, the cyanobacterial CAT and POD activities and the contents of GSH and AsA increased quickly to 309.8 and 786.0%, 183.5 and 246.0% of the controls (Figure 4), respectively, indicating that H_2_O_2_ probably can be decomposed into nontoxic H_2_O and O_2_ quickly. Therefore, H_2_O_2_ induced by CBSL or from O_2_^−^ probably contributed lesser to MDA production in cyanobacterial cell.

MCs pose a serious threat to human and environmental health, and are one of the most significant problems brought by *Microcystis* water blooms. Moreover, the cyanobacteria can release massive toxins under the stresses of algicides, which aggravates water-quality problems (Griffiths and Saker, 2003; Ross et al., 2006). Previously studies also found that exposure to algicidal compounds from *Streptomyces*, such as 2′-deoxyadenosine (Zhang et al., 2015), L-valine (Zhang et al., 2016a) tryptamine, and tryptoline (Zhang et al., 2016b), would stimulate *Microcystis* to produce and release more MC-LR within a certain period of cultivation time. These MCs are chemically stable and biological degradation plays an important role in detoxification of the MCs in natural water environments (Dawson, 1998). Many microorganisms can degrade cyanobacterial toxins, such as *Sphingomonas*, *Arthrobacter*, *Bacillus* (Ling et al., 2024), *Lactobacillus plantarum* IS-10506 and IS-20506, *Lactobacillus rhamnosus* GG and LC-705, *Bifidobacterium lactis* 420 and Bb12, *Bifidobacterium longum* 46 (Nybom et al., 2007a; Nybom et al., 2007b; Nybom et al., 2008), *Burkholderia* (Lemes et al., 2008), *Poterioochromonas* (Ou et al., 2005), *Paucibacter toxinivorans* (Rapala et al., 2005) and *Sphingosinicella microcystinivorans* (Maruyama et al., 2006). Moreover, MCs could be degraded effectively by mixed bacterial populations (Babica et al., 2005; Bourne et al., 2006; Edward et al., 2008). In our test, the contents of MC-RR and MC-LR decreased by 86.7 and 81.0% for treatment of CBSL, and by 96.3 and 89.9% for treatment of mycelia (Figure 5). Therefore, the additions of both CBSL and mycelia can significantly promote the degradations of MCs (*p* < 0.01). *Microcystis* has many nonculturable attached bacteria, such as *Lactobacillus*, *Sphingomonas*, and *Bifidobacterium* etc. (Xiao et al., 2024; Wang et al., 2024). These nonculturable attached bacteria were probably involved in the degradations of MCs in this study.

RAs, strong animal teratogens (Bryant and Gardiner, 1992), are usually known as endogenous metabolites of vitamin A in vertebrate animals. However, Wu et al. (2012) found that RAs can be produced by most of the cyanobacteria. This finding would make people reconsider hazards of cyanobacterial blooms. The exogenous RAs from cyanobacteria are probably one of the main reasons of deformed animals in eutrophic water. Drinking water contaminated by the exogenous RAs may cause the deformity of human embryo. Therefore, how to remove the exogenous RAs in water environments in time should be emphasized when we control the cyanobacterial blooms. In this study, RAs were not detected by HPLC after *M. aeruginosa* FACHB-905 culture being treated with both CBSL and mycelia, indicating that RAs were effectively removed by strain LMJ-114 and its metabolites.

## 5 Conclusion

*Streptomyces* LMJ-114 showed good algicidal activity on both *Microcystis* and filamentous cyanobacteria. Its metabolites influenced *M. aeruginosa* antioxidant system significantly, and resulted in the formation of abundant ROS, and a resultant serious lipid-peroxidation radical chain reaction, and subsequent a sunken and perforated cell surface. L-valine is one of its algicidal compounds. Application of strain LMJ-114 and its metabolites in *M. aeruginosa* culture greatly reduced MCs and RAs. Strain LMJ-114 and its metabolites showed good potential application in controlling cyanobacterial blooms.

## Data Availability

The 16S rRNA gene sequence of strain LMJ-114 has been deposited in GenBank under accession number PV915256 and is available via the NCBI database (https://www.ncbi.nlm.nih.gov/nuccore/). Additional data are provided in the published article and its supplementary materials, or can be obtained from the authors upon request.
