# Supplementary material for: Algicidal activity of Streptomyces sp. LMJ-114 against Microcystis aeruginosa
Source: Front Microbiol. 2025 Nov 12;16:1669970. doi: 10.3389/fmicb.2025.1669970 (PMC12657015; doi:10.3389/fmicb.2025.1669970)
Supplement: Supplementary file 1 [file Data_Sheet_1.pdf]

# Algicidal activity of *Streptomyces* sp. LMJ-114 against *Microcystis aeruginosa*

Mijia Du[1¶], Qian Xie[1¶], Hongqiu Shi[1*], Jianyuan Yang[1], Qigen Guo[1], Yuqin Zhang[2*] and Binghuo Zhang[1*]

[1] College of Pharmacy and Life Sciences, Jiujiang University, Jiujiang, China, [2] Institute of Medicinal Biotechnology, Chinese Academy of Medical Sciences & Peking Union Medical College, Beijing, China

**Author for correspondence:**

**Hongqiu Shi**

Tel & Fax: +86-792-8552912

E-Mail: 446080626@qq.com

**Yuqin Zhang**

Tel & Fax: +86-10-83167110

E-Mail: zhyuqin@126.com (Y. Zhang)

**Bing-Huo Zhang**

Tel & Fax: +86-792-8552912

E-Mail: binghuozh@126.com

Mijia Du, Qian Xie contributed equally to this work.

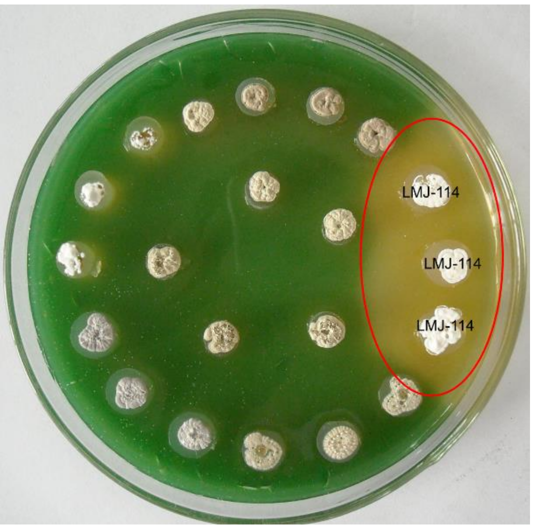


**Fig. S1** Algicidal activity of strain LMJ-114 on *Microcystis aeruginosa* FACHB-905 lawn

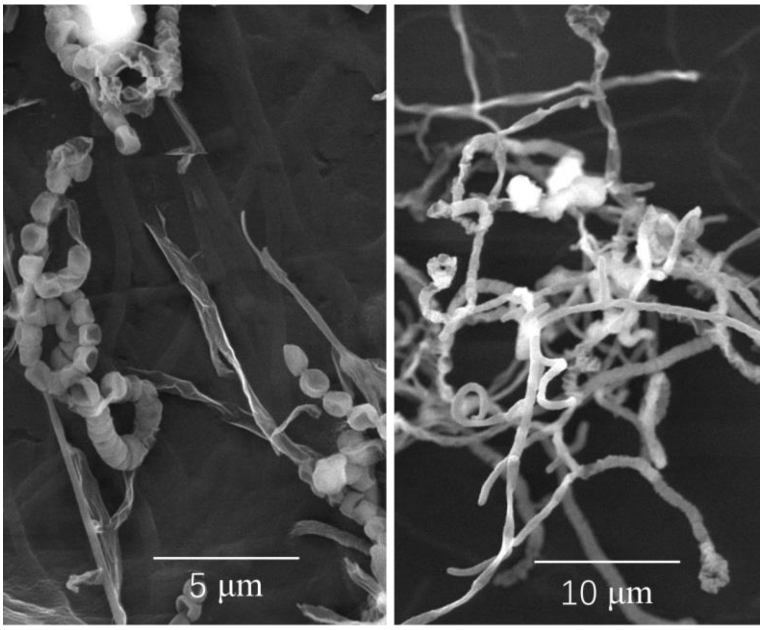


**Fig. S2** Scanning electron microscope photograph of strain LMJ-114

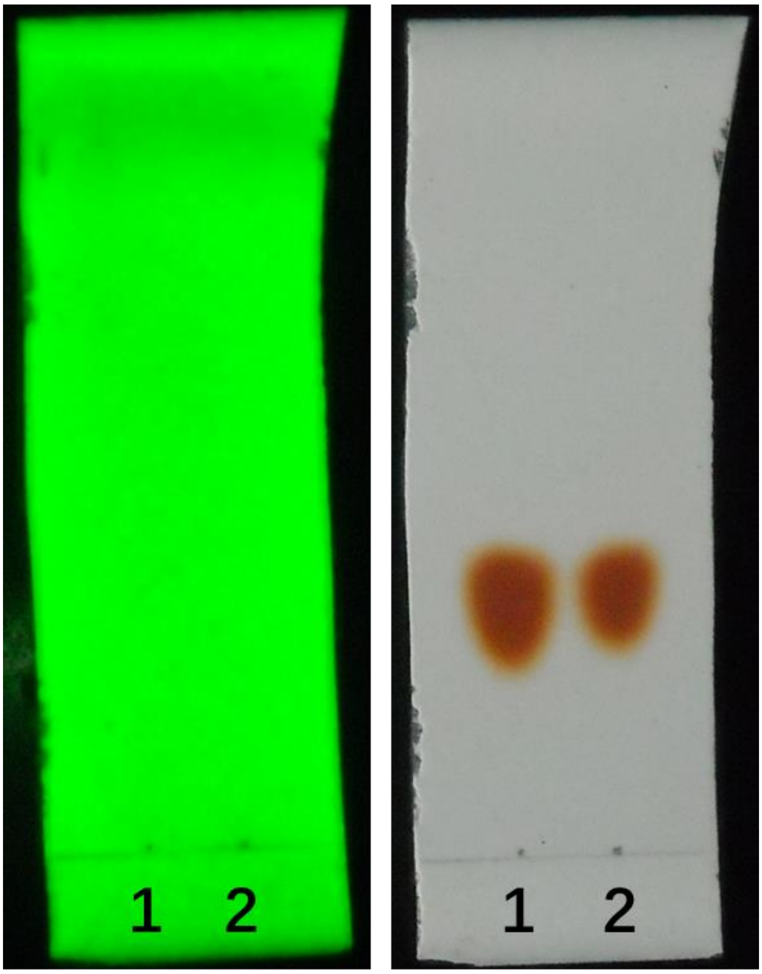


**Fig. S3** Results of thin layer chromatography using chromatographic solvent system consisted of n-butyl alcohol/acetic acid/water (4/1/5; v/v/v). Compound 1, isolated from LMJ-114; compound 2, L-valine

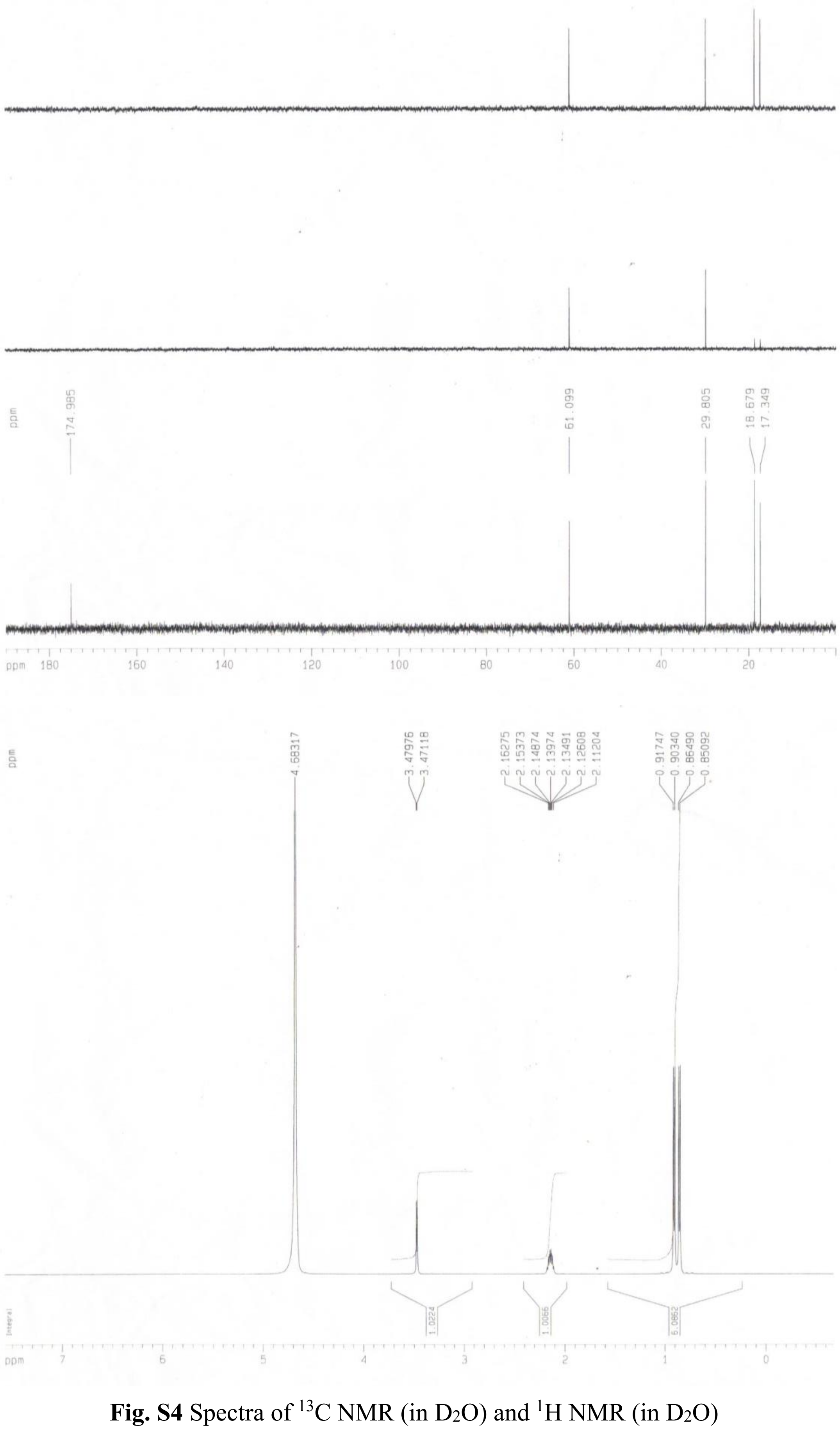


**Fig. S4** Spectra of $^{13}C$ NMR (in $D_2O$) and $^{1}H$ NMR (in $D_2O$)
